# Juvenile Inflammatory Rheumatism Real-Life Clinical Practice Strategies (JIR-CliPS) network: methodology and the development process

**DOI:** 10.1186/s13023-026-04276-8

**Published:** 2026-04-09

**Authors:** Helmut Wittkowski, Raffaella Carlomagno, Sophie Georgin-Lavialle, Cristina Costa Duarte Lanna, Dzifa Dey, Marie Frank, Daiva Gorczyca, François Hofer, Tilmann Kallinich, Sylvia Kamphuis, Phil Riley, Mariana Rodrigues, Sezgin Sahin, Ausra Snipaitiene, Katerina Theodoropoulou, Natasa Toplak, Jacqueline Yan, Michaël Hofer, Véronique Hentgen

**Affiliations:** 1 Department of Pediatric Rheumatology and Immunology, University Hospital Münster, Münster, Germany; 2 Pediatric Rheumatology, Immunology and Allergology Unit, Department of Pediatrics Lausanne, University Hospital of Lausanne, University of Lausanne, Vaud, Switzerland; 3 DMU 3ID, Internal Medicine Department, Tenon Hospital, AP-HP and ERN RITA, Sorbonne University, Paris, France; 4 French National Reference Center of Autoinflammatory Diseases and Inflammatory Amyloidosis, Paris, France; 5 Locomotor Apparatus Department, Medical School, Universidade Federal of Minas Gerais, Belo Horizonte, Brazil; 6 Department of Medicine and Therapeutics, College of Health Sciences, University of Ghana Medical School, Korle-Bu, Accra, Ghana; 7 Fondation Rhumatismes Enfants Suisse, Lausanne, CH-1000 Switzerland; 8 Center for Chronically Sick Children, Department of Paediatric Respiratory Medicine, Immunology and Critical Care Medicine, Charité – Universitätsmedizin Berlin, Freie Universität Berlin and Humboldt- Universität Berlin zu Berlin, Berlin, Germany; 9 Department of Paediatric Respiratory Medicine, Immunology and Critical Care Medicine, Charité Universitätsmedizin, Berlin, Germany; 10 German Center for Child and Adolescent Health (DZKJ), Partner Site Berlin, Berlin, Germany; 11 Deutsches Rheuma-Forschungszentrum (DRFZ), an Institute of the Leibniz Association, Berlin, Germany; 12 Pediatric Rheumatology, Sophia Children’s Hospital, Erasmus University Medical Center, Rotterdam, Netherlands; 13 Royal Manchester Children’s Hospital, Manchester, UK; 14 Pediatric Rheumatology, Centro Hospitalar de São João, Porto, Portugal; 15 Department of Pediatric Rheumatology, Istanbul University-Cerrahpasa, Istanbul, Turkey; 16 Department of Pediatrics, Lithuanian University of Health Sciences, Kaunas, Lithuania; 17 Faculty of Medicine, Department of Allergology, Rheumatology and Clinical Immunology Ljubljana, UCH, UMC Ljubljana, Ljubljana, Slovenia; 18 Paediatric Rheumatology, Starship Child Health, University of Auckland, Auckland, New Zealand; 19 Department of Paediatrics, Hôpital Riviera-Chablais, Rennaz, CH-1847 Switzerland; 20 Department of General Paediatrics, French Reference Centre for Autoinflammatory diseases and amyloidosis (CEREMAIA), Versailles Hospital, Le Chesnay, France

**Keywords:** Arthritis, Juvenile, Vasculitis, Still’s disease, Lupus nephritis, Familial mediterranean fever

## Abstract

**Background and objective:**

Juvenile-onset Inflammatory Rheumatisms (JIR) are rare systemic inflammatory diseases that begin in childhood or adolescence. Numerous recommendations have been developed to support physicians, but implementation may vary in real-life. The JIR-Clinical Practice Strategies (CliPS) project investigates clinical practices for JIR diseases. This article describes the rationale of the JIR-CliPS project to serve as methodological foundation for future publications on disease-specific findings.

**Methods:**

The JIR-CLiPS network was launched in 2020 to collect CliPS in real-life on five medical conditions: lupus nephritis, vasculitis (including IgA vasculitis and Kawasaki disease), use of biologicals for hereditary autoinflammatory diseases, periodic fever with aphthous stomatitis, pharyngitis and adenitis (PFAFA) / systemic undefined recurrent fevers (SURF) syndrome, and Still’s disease. Specific questionnaires were developed, covering physician demographics, disease-specific approaches, and local healthcare constraints.

**Results:**

From June 2022 to April 2024, 534 physicians from 66 countries across six continents participated, completing in 1,193 questionnaires. Respondents were primarily pediatricians and rheumatology specialists, working both at universities and in local hospitals; although most participants reported at least 10 years’ experience, younger doctors also took part. Participants from only a few countries shared the development of local or national JIR-specific recommendations, with variable awareness and adherence.

**Conclusion:**

The JIR-CliPS network has achieved broad international physician participation. Thanks to the knowledge gathered, a library with CliPS adapted to various local and country-specific constraints is currently under development, bridging the gap between published recommendations and achievable everyday practice. This robust methodology ensures a comprehensive and globally representative dataset for future disease-specific publications.

## Introduction

Juvenile-onset inflammatory Rheumatic diseases (JIR) constitute a group of rare autoimmune and autoinflammatory diseases that begin in childhood or adolescence. Physicians treating JIR patients face a dual challenge: the available literature on JIR is limited, and these are systemic diseases characterized by highly variable phenotypic expressions and diagnoses that rely primarily on clinical criteria. Over the years international recommendations have been developed and implemented to assist physicians in the management of these rare and challenging diseases. However, adherence to clinical guidelines is a well-documented challenge in healthcare. Compliance rates for non-rare diseases are reported to range between 30 and 70% [1–3]. This suboptimal adherence has been shown to be linked to several factors, including a lack of awareness regarding the existence of guidelines, gaps in physician knowledge, disagreement with the guidelines, and deficiencies in leadership and teamwork within clinical settings. For rare pediatric rheumatological diseases, it seems reasonable to assume that practical application of expert recommendations and guidelines is likely to be even lower [4]. 

The JIR-Network (JIRcohorte.org) is an international community of healthcare providers caring for pediatric and adult patients suffering from JIR [5]. The JIR-clinical practice strategies (CliPS) project was developed due to a high variability of patient management in the network, including poorly defined indications for using IL-1 inhibitors in monogenic recurrent fevers [6]. We hypothesized that the recommendations developed for different JIR diseases are not universally applied due to the diversity of medical systems and financial resources across countries and that the dissemination of physicians’ authentic experiences within the medical community may enhance patient outcomes for JIR conditions. The JIR-CliPS network has set up a consortium of physicians with four main objectives: (i) to document routine clinical care practices; (ii) to analyze the factors that contribute to variations in physicians’ practices; (iii) to develop clinical practice strategies based on questionnaire responses; and (iv) to organize educational events to bridge knowledge gaps.

This article describes how the JIR-CliPS project was developed and implemented and presents the characteristics of the participants.

## Methods

### The creation of the network

The JIR network was established in 2013 centered around two key initiatives: the JIR Cohort, an international real-life registry for juvenile inflammatory rheumatisms, and the JIR Academy, which organizes educational events for physicians caring for these patients. Over the years, colleagues from 89 countries across six continents have joined the network contributing to its research and educational activities. Insights gained from the registry’s published research, along with discussions at educational events, highlighted the need to evaluate patient management practices in real-world settings across diverse contexts.

Aligned with the expertise and interests of the network’s key contributors, we have selected the following five medical conditions: (1) Lupus nephritis (LN), (2) Vasculitis including Immunoglobulin A vasculitis (IgA-V) and Kawasaki disease (KD), (3) Use of biological drugs for treatment of hereditary autoinflammatory diseases (bAID), (4) Periodic fever with aphthous stomatitis, pharyngitis and adenitis (PFAFA) and systemic undefined recurrent fevers (SURF) syndrome, (5) and Still’s disease. The JIR cohort research group invited physicians from various countries, mostly pediatric rheumatologists active in the Pediatric Rheumatology European Society (PReS), and the project was endorsed by PReS working parties (vasculitis, lupus, autoinflammation). Other collaborators are adult physicians, experts for autoinflammatory diseases and Still’s disease, and the chairmen of the PReS WPs and of the Eurofever network.

**Fig. 1:**
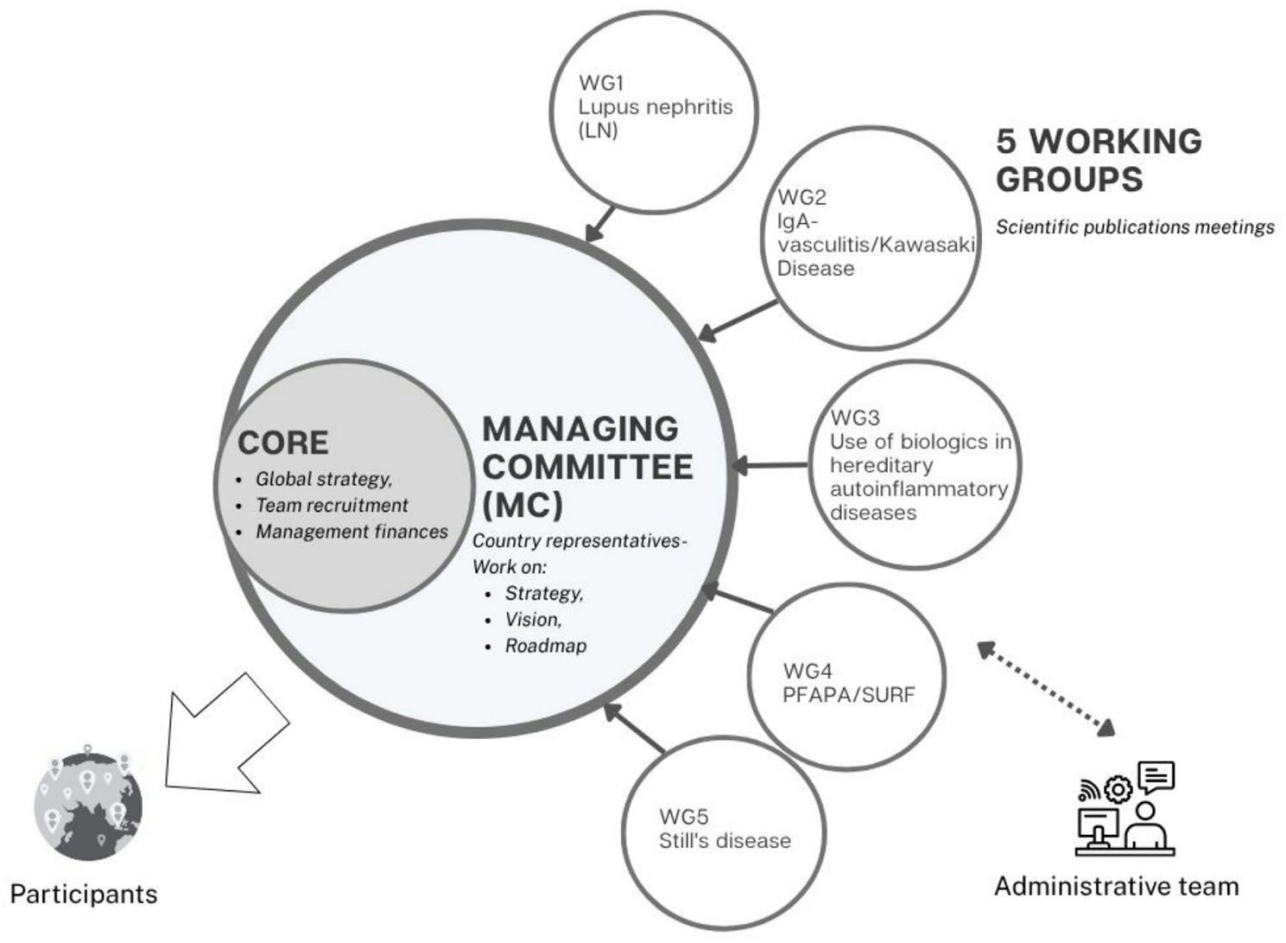
JIR CliPS network organization

The acquisition of the European Cooperation in Science and Technology (COST) funding in 2021 permitted to extend the project to additional countries. During the Kick-off meeting (October 2021), we defined Working Groups (WGs), that prepared questionnaires on each of the five medical conditions. Subsequent meetings refined the questionnaires to ensure their relevance and effectiveness. Figure 1 illustrates the organization of JIR CliPS network.

### Capturing the real-life practice: elaboration and evaluation of the questionnaires

The five questionnaires in English had a common structure that included the following parts: (a) a one-page introduction explaining the project and the aim of the questionnaire including the information that the answers would not be judged as right or wrong, (b) demographic questions about the physician completing the questionnaire, (c) disease-specific questions, (d) local or national constraints in the treatment of the specific diseases, and (e) questions related to the COVID-19 pandemic. In all questionnaires, parts b, d and e were harmonized among the five groups, while part c represented the main section with disease-specific questions. The disease-specific section developed by each WG in the first version of the questionnaire included 93 questions for LN, 49 for IgA-V and KD, 132 for (bAID), 146 for PFAPA/SURF, and 67 for Still’s disease.

Internal validation was ensured by independent reviews. Following internal validation, we assessed the technical functionality and clarity of the questionnaires through a round of interviews conducted within the five WGs. After this process, the questionnaires, developed on the Eval&Go^®^ online platform, were finalized for dissemination. In the next step an evaluation of the first version of the questionnaires through a pilot study was conducted from June to September 2022. This pilot study, supported by funding from the World University Network (WUN), was carried out by teams working in diverse healthcare settings. Four pediatric rheumatology centers affiliated with WUN member universities participated: Accra (Ghana), Auckland (New Zealand), Belo Horizonte (Brazil), and Lausanne (Switzerland).

### Enrollment process and data analysis

**Fig. 2:**
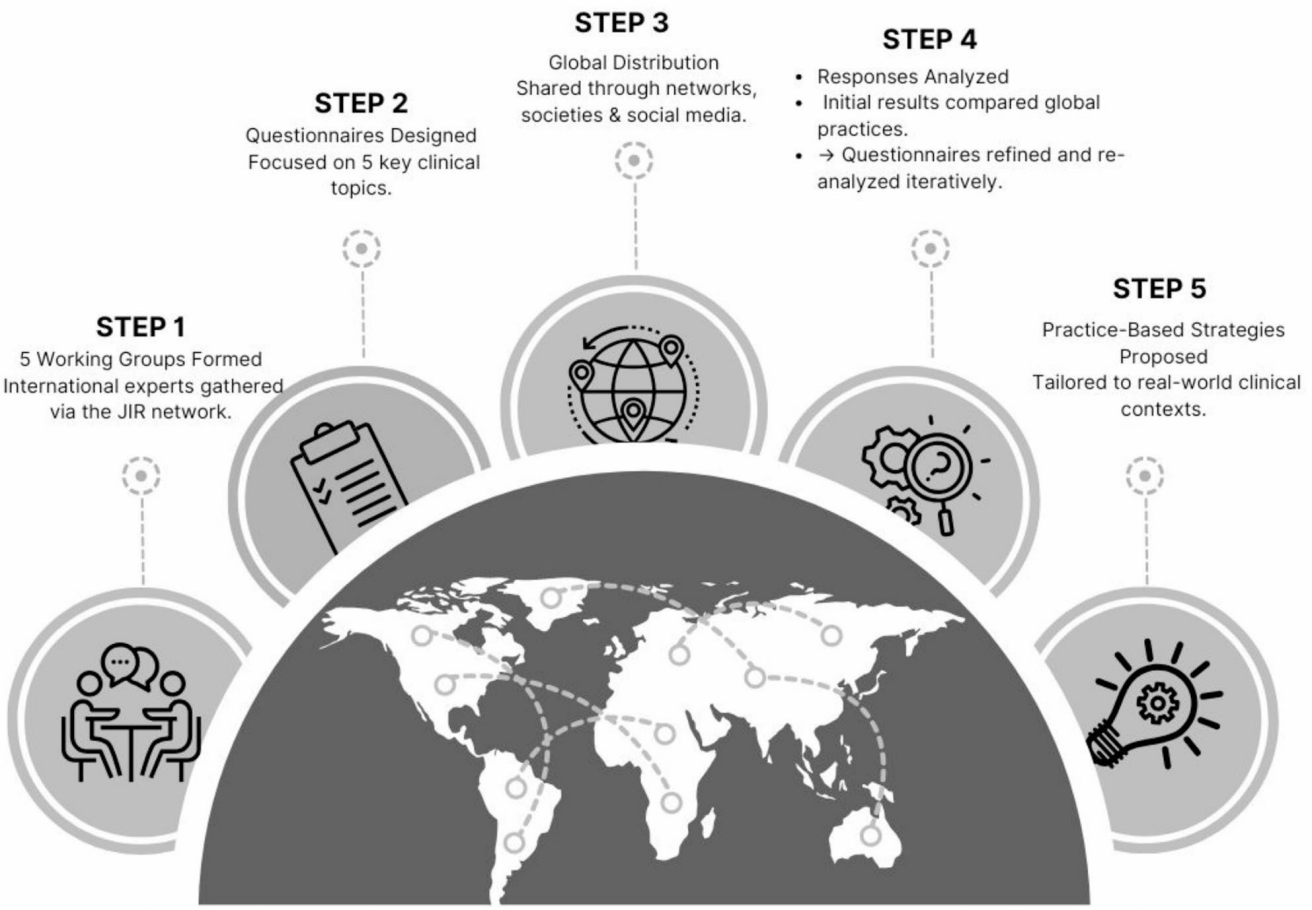
JIR CLiPS steps

Starting in January 2023, the finalized version of the five questionnaires was distributed to interested physicians worldwide. All members of the JIR Network were contacted to participate to the survey. Recruitment of participants was also initiated through national and international societies for pediatric rheumatology and for the specialties concerned by the project (nephrology, immunology, cardiology, internal medicine, pediatrics). Based on the initial data collection from the questionnaires (March 2023), each WG analyzed the responses extracting the CliPS in flowcharts reflecting the different approaches. All WGs had to rework certain questions to better capture the CliPS. Each WG defined its own methodology and statistical analyses to describe the clinical strategies and flowcharts, as these depended on the specific questions formulated in each questionnaire. The main steps of the JIR CLiPS project are outlined in Fig. 2.

### Ethical considerations

This study involved the distribution of an online questionnaire as part of the JIR-CliPS project. Participants provided informed consent by voluntarily completing and submitting the questionnaire. They also agree to the processing of their personal data by the Fondation RES, which is the data processor of the CliPS project. The study was conducted in accordance with the Declaration of Helsinki and applicable data protection regulations, including the General Data Protection Regulation (GDPR). Ethics approval was deemed unnecessary due to the non-invasive nature of the research and the absence of sensitive personal data collection.

## Results

### Description of the respondents’ characteristics (cf. Table 1 and Fig. 3)

**Table 1:** Demography of the responders

	Overall	Lupus nephritis	IgA vasculitis and Kawasaki disease	Biologic drugs in autoinflammatory diseases	PFAPA/SURF	Still’s disease
Total number of responders	534	151	252	200	273	317
Gender (N, %)						
Female Male No answer	362 (67.8) 169 (31.6) 3 (0.6)	102 (68.5) 47 (31.5) 2 (1.3)	184 (74.5) 66 (26.7) 2 (0.8)	136 (70.1) 62 (32.0) 2 (1.0)	192 (71.9) 79 (29.6) 2 (0.7)	207 (67.0) 107 (34.6) 3 (1.0)
Specialty (N, %)						
Pediatric Adult Pediatric + Adult Rheumatology Immunology Nephrology Cardiology Infectious Disease Internal Medicine Primary care Other^a^ > 1 specialty No answer	398 (74.5) 100 (18.7) 25 (4.7) 390 (73.0) 77 (14.4) 30 (5.6) 20 (3.7) 14 (2.6) 14 (2.6) 84 (15.7) 11 (2.1) 121 (22.7) 11 (2.1)	121 (80.1) 20 (13.2) 9 (6.0) 128 (84.8) 14 (9.3) 15 (9.9) 1 (0.7) 0 1 (0.7) 14 (9.3) 0 30 (5.6) 1 (0.7)	218 (86.5) 15 (6.0) 13 (5.2) 178 (70.6) 27 (10.7) 15 (6.0) 16 (6.3) 6 (2.4) 0 65 (25.8) 4 (1.6) 65 (25.8) 6 (2.4)	161 (80.5) 26 (13.0) 11 (5.5) 171 (85.5) 39 (19.5) 8 (4.0) 2 (1.0) 2 (1.0) 5 (2.5) 24 (12.0) 4 (2.0) 62 (31.0) 3 (0.6)	232 (85.0) 26 (9.5) 12 (4.4) 222 (81.3) 46 (16.8) 5 (1.8) 2 (0.7) 11 (4.0) 3 (1.1) 37 (13.6) 3 (0.6) 64 (23.4) 3 (1.1)	215 (67.8) 83 (26.2) 16 (5.0) 274 (86.4) 47 (14.8) 9 (2.8) 6 (1.9) 2 (0.6) 12 (3.8) 33 (10.4) 6 (1.9) 80 (25.2) 3 (1.0)
Type of institution (N, %)^b^						
University Tertiary Center Hospital Private practice Other No answer	337 (63.1) 77 (14.4) 90 (16.9) 15 (2.8) 8 (2.2) 3 (0.6)	97 (64.2) 28 (18.5) 20 (13.2) 4 (2.6) 2 (1.3) 0	149 (59.1) 343 (17.1) 49 (19.4) 6 (2.4) 4 (1.6) 1 (0.4)	131 (67) 28 (14.0) 31 (15.5) 4 (2.0) 5 (2.5) 1 (0.5)	167 (61.2) 38 (13.9) 53 (19.4) 9 (3.3) 5 (1.8) 4 (1.5)	208 (65.6) 50 (15.8) 44 (13.9) 7 (2.2) 6 (1.9) 2 (0.6)
Type of care (N, %)^c^						
Outpatients Inpatients Both No answer	21 (3.9) 8 (1.5) 500 (93.6) 5 (0.9)	4 (2.6) 1 (0.7) 145 (96.0) 1 (0.7)	7 (2.8) 2 (0.8) 242 (96.0) 1 (0.4)	8 (4.0) 2 (1.0) 189 (94.5) 1 (0.5)	14 (5.1) 1 (0.4) 256 (93.8) 2 (0.8)	10 (3.2) 2 (0.6) 303 (95.6) 2 (0.6)
Type of patients (N, %)^d^						
Children Adults Both No answer	387 (72.5) 72 (13.5) 69 (12.9) 6 (1.1)	116 (76.8) 15 (9.9) 19 (12.6) 1 (0.7)	211 (83.7) 7 (2.8) 32 (12.7) 2 (0.8)	151 (75.5) 19 (9.5) 29 (14.5) 1 (0.5)	225 (82.4) 16 (5.9) 30 (11.0) 2 (0.8)	208 (65.6) 61 (19.2) 46 (14.5) 2 (0.7)
Years of practice (N, %)^e^						
0–4 years 5–9 years ≥ 10 years No answer	102 (19.1) 158 (29.6) 268 (50.2) 6 (1.1)	24 (15.8) 53 (35.1) 74 (49.0) 0	47 (18.6) 79 (31.3) 125 (49.6) 1 (0.4)	36 (18.0) 65 (32.5) 98 (49.0) 1 (0.5)	40 (14.7) 93 (34.0) 138 (50.5) 2 (0.8)	49 (15.4) 100 (21.6) 164 (51.7) 4 (1.3)

^a^ Other included: Dermatology, Hemato-oncology, Clinical Genetics, Gastroenterology, Allergology

^b^ Some participants gave multiple responses: we used the most academic one (University> Tertiary> Hospital> Private)

^c^ 5 missing responses

^d^ 5 missing responses

^e^ 6 missing responses

**Fig. 3:**
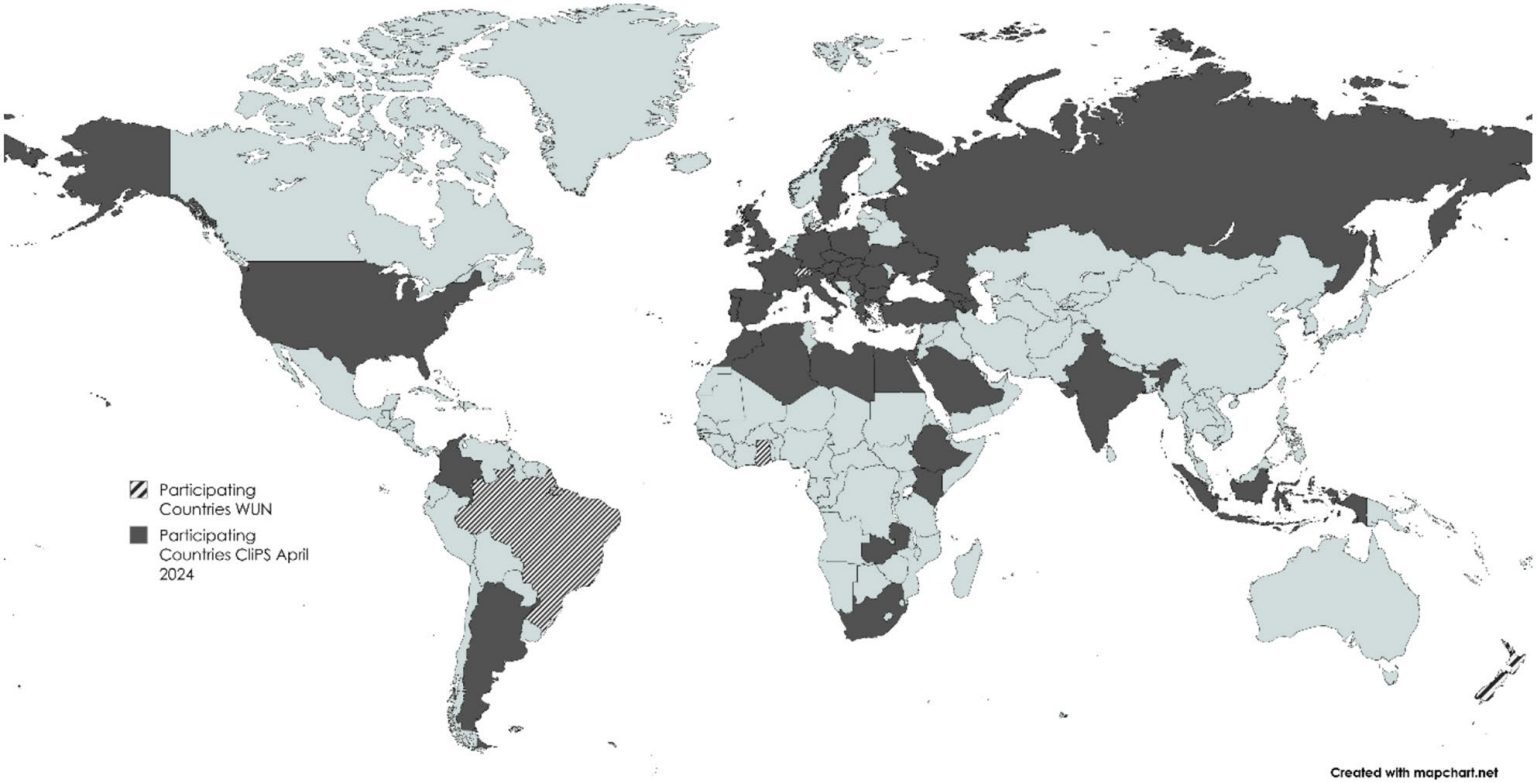
Participating Countries JIR & CliPS JIR WUN – April 2024

Up to April 2024, 534 physicians originated from 66 countries on 6 continents participated to the study: 31 countries in Europe, 14 in Africa, 11 in Asia, 5 in South America, 4 in North America and one in Oceania (Fig. 1). Countries’ participation varied according to the medical conditions: LN: 38, IgA-V and KD: 52, bAID: 45, PFAPA/SURF: 49, and Still’s disease: 56. The number of participants per country ranged from 1 to 68, and seven countries had more than 20 participants (France, Turkey, Brazil, Switzerland, Spain, United Kingdom and Germany). Most participants were female (362), saw both inpatients and outpatients (500) and worked in a university hospital (337), but physicians working in a local hospital (90) or in private practice (15) participated also in the survey. Around half of the participants reported having at least 10 years’ experience in caring for patients with the diseases concerned by the project. Three hundred ninety-eight participants were pediatricians and 390 declared rheumatology as their specialty; according to the questionnaire, other specialties were mentioned: immunology or internal medicine (91), primary care (84), nephrology (30), cardiology (20) and infectious diseases (14). 387 physicians treated only pediatric patients, 69 both adult and pediatric patients.

### Description of the questionnaires’ characteristics (cf. Table 2)

**Table 2:** Country specificity of the answered questionnaires

	Overall	LN	IgA-V and KD	bAID	PFAPA/SURF	Still
Total number of questionnaires	1193	151	252	200	273	317
Country (N, %)						
Africa Algeria Libya Ghana Tunisia	102 (8.5) 25 20 16 12	14 (7.0)	15 (9.9)	12 (4.4)	33 (10.4)	28 (11.1)
America North Mexico United States	31 (2.6) 12 11	5 (2.5)	2 (1.3)	10 (3.7)	7 (2.2)	7 (2.8)
America South Brazil	154 (12.9) 141	28 (14.0)	31 (20.5)	34 (12.5)	32 (10.1)	29 (11.5)
Asia Israel India	74 (6.2) 28 18	16 (8.0)	6 (4.0)	17 (6.2)	20 (6.3)	15 (6.0)
Europa Turkey France Switzerland United Kingdom Germany Spain Croatia Portugal Italy Netherlands	781 (65.5) 173 118 58 57 46 39 38 37 30 21	131 (65.5)	90 (59.6)	188 (68.9)	218 (68.8)	154 (61.1)
Oceania New Zealand	51 (4.3) 51	6 (3.0)	7 (4.6)	12 (4.4)	7 (2.2)	19 (7.5)
Country GDP (N, %)^a^						
high upper middle lower middle low	686 (57.5) 477 (40.0) 28 (2.3) 2 (0.2)	67 (44.4) 83 (55.0) 0 (0) 1(0.7)	144 (57.1) 108 (42.9) 0 (0) 0 (0)	113 (56.5) 87 (43.5) 0 (0) 0 (0)	169 (61.9) 95 (34.8) 9 (3.3) 0	193 (60.9) 104 (32.8) 19 (6.0) 1 (0.3)
Awareness of national recommendations (N, %)^b^		**NA**				**NA**
Disease Countries (N) Yes No No answer			**IgA-V** 13 26 76 150	**CAPS** 10 24 48 128	**PFAPA** 3 10 54 209	
Disease Countries (N) Yes No No answer			**KD** 22 77 91 84	**FMF** 9 30 57 113		

^a^ https://blogs.worldbank.org/en/opendata/new-world-bank-group-country-classifications-income-level

^b^ number of countries where national recommendations were reported

^c^ number of participants

A total of 1193 questionnaires were completed, including 151 for LN (13%), 252 for IgA-V and KD (21%), 200 for bAID (17%), 273 for PFAPA/SURF syndromes (23%), and 317 for Still’s disease (27%). Three countries provided more than 100 questionnaires each corresponding to a third of the 1193 responses: Turkey 173, Brazil 141 and France 118. Most participants came from a country with a high gross domestic product (GDP), but we also had respondents from countries with a lower-middle or low GDP. The same GDP distribution was observed for participants in the different questionnaires, with the exception of LN, where most respondents came from countries with upper-middle GDP.

In 3/5 questionnaires (bAID, IgA-V and KD, and PFAPA/SURF), questions were asked about awareness of local or national recommendations for the specific medical condition. Recommendations were reported in 10/45 (22%) countries for CAPS, 9/45 (20%) for FMF, 13/52 (25%) for IgA-V, 22/52 (42%) for KD, and 3/49 (6%) for PFAPA [7–33]. For SURF, no recommendations were mentioned by the participants. As shown on Table 2, only a minority of the participants were aware of the existence of recommendations. For countries where one or more respondents mentioned a national recommendation, only 26% mentioned its existence, 18% said they were not aware of any recommendations for the disease in question in their country, and 56% did not answer the question. Awareness of national recommendations is highest in France and New Zealand, and lowest in Brazil; it is highest for KD and lowest for PFAPA.

## Discussion

To the best of our knowledge, the JIR-CliPS project is the first initiative to systematically collect and analyze real-world clinical strategies for managing rare juvenile-onset inflammatory rheumatisms (JIR) on a global scale. These diseases are characterized by their rarity and clinical heterogeneity, and most are supported by only low-grade, expert-based recommendations. Furthermore, significant variability in healthcare infrastructures and access to therapies across countries has resulted in disparate clinical practices, as illustrated by national adaptations or omissions of international guidelines [34].

Conditions such as PFAPA and undefined recurrent fevers (e.g., SURF) are often absent from formal recommendations, and even when guidelines exist, they tend to focus on prototypical cases, overlooking the spectrum of presentations encountered in real-life practice. To address this, we developed five condition-specific questionnaires to document daily decision-making across settings. In the absence of validated tools for this purpose, each Working Group piloted and refined its approach to ensure that the questionnaires could effectively capture clinical variation and identify emerging strategies.

Our hypothesis is that disseminating these real-world approaches can support evidence-informed yet adaptable care models, ultimately improving patient outcomes for JIR conditions. The richness of the data offers an opportunity to develop a library of clinical strategies that reflect not just theoretical standards, but practical, achievable care worldwide.

### A global perspective on practices and gaps in knowledge

Although responses from European countries were more frequent, physician participation from 66 countries across six continents provides valuable insight into the management of JIR under diverse health system constraints. Notably, 20% of respondents practiced outside university or tertiary referral centers—settings often underrepresented in clinical trials—shedding light on approaches used in secondary hospitals or private practice.

One of the most striking findings is the limited awareness of national guidelines: only 25% of respondents could identify recommendations for the diseases studied. This highlights a significant knowledge gap and raises important questions about the dissemination and accessibility of existing guidelines. While this study was not designed to analyze these barriers in depth, ongoing disease-specific analyses may uncover contributing factors such as language barriers, limited continuing education, or lack of centralized guidance from professional bodies.

The diversity in specialties, levels of experience, and care settings among respondents provides a rare and rich foundation for evaluating how knowledge is translated into practice. It also underscores the need for flexible, context-sensitive strategies that reflect local realities rather than universal assumptions.

### Toward contextualized recommendations: bridging evidence and feasibility

The variability in practice observed in this study mirrors findings from broader health services research: evidence-based recommendations often fail to consider practical constraints such as drug availability, health policy limitations, or socioeconomic disparities [4]. As such, many physicians—particularly in resource-limited settings—must rely on experience or modified protocols rather than formal guidelines.

This disconnect has real implications. For example, studies of childhood-onset lupus from centers in the United States, Brazil, and India have shown that recommended standards of care are not consistently met across settings, with downstream consequences for disease outcomes [35]. JIR-CliPS aims to illuminate these “gray zones” of practice and collect strategies that, though not formally endorsed, may be clinically effective and contextually appropriate.

**Fig. 4:**
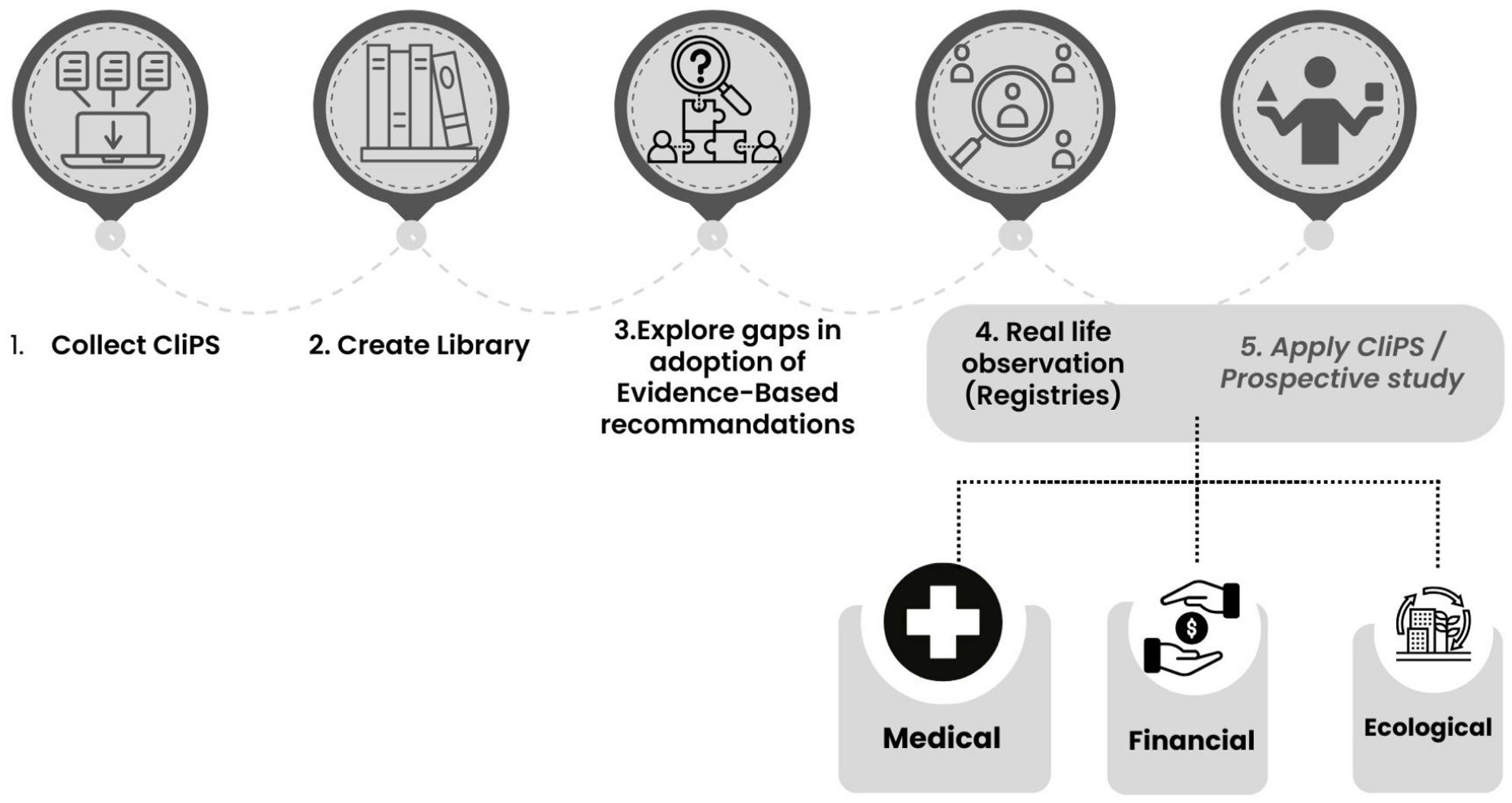
JIR CliPS vision

A future goal of the CliPS initiative is to evaluate the strategies collected through multiple lenses:

- **Patient outcomes**: Comparative effectiveness research can determine whether these strategies translate into improved health metrics.
- **Cost-effectiveness**: Economic evaluations will help assess which approaches offer the most benefit per resource invested.
- **Feasibility and sustainability**: The environmental and logistical impact of clinical practices should also be considered, particularly in global health planning. The overall vision for the JIR CliPS project is illustrated in Fig. 4.

## Conclusion and future directions

The JIR-CliPS network has successfully engaged a diverse, international cohort of physicians, generating a globally representative dataset that reflects the complexity of real-world JIR care. This initiative demonstrates that clinical expertise, even in the absence of robust evidence or uniform guidelines, can be harnessed and systematically documented.

Moving forward, the CliPS data will be used to develop condition-specific clinical practice strategies that are tailored to the realities of different countries and care settings. By bridging the gap between evidence-based ideals and day-to-day feasibility, this project has the potential to inform future guideline development, continuing education, and international consensus-building—ultimately improving care for children and adolescents with rare systemic inflammatory diseases around the world.

## Data Availability

The datasets used and/or analyzed during the current study are available from the corresponding author on reasonable request.
